# A qualitative study of e-cigarette emergence and the potential for renormalisation of smoking in UK youth

**DOI:** 10.1016/j.drugpo.2019.11.006

**Published:** 2020-01

**Authors:** R. Brown, L. Bauld, E. de Lacy, B. Hallingberg, O. Maynard, J. McKell, L. Moore, G. Moore

**Affiliations:** a Centre for the Development and Evaluation of Complex Interventions for Public Health Improvement, Cardiff University, UK; b Usher Institute of Population Health Sciences & Informatics, University of Edinburgh, UK; Director, SPECTRUM Consortium; c Department of Applied Psychology, Cardiff Metropolitan University; d MRC Integrative Epidemiology Unit (IEU) at the University of Bristol, Bristol, UK/UK Centre for Tobacco and Alcohol Studies (UKCTAS) and School of Psychological Science, University of Bristol, Bristol, UK; e Institute for Social Marketing, University of Stirling and UK Centre for Tobacco and Alcohol Studies, UK; f MRC/CSO Social and Public Health Sciences Unit, University of Glasgow, UK; g Centre for the Development and Evaluation of Complex Interventions for Public Health Improvement, Cardiff University, UK; SPECTRUM Consortium

**Keywords:** E-cigarette, Renormalisation, Youth, Tobacco

## Abstract

**Background:**

Growth of e-cigarette use among smokers has raised concerns over uptake by non-smokers, particularly young people. Legislative changes aimed in part at reducing youth exposure to e-cigarettes include the EU Tobacco Products Directive (TPD). A core justification for such measures is the belief that e-cigarettes can lead to tobacco smoking through mechanisms of renormalisation including: mimicking and normalizing the act of smoking; increasing product acceptability via marketing; nicotine exposure. These mechanisms are here explored in relation to findings from qualitative research.

**Methods:**

This paper reports results from twenty-one group interviews with 14–15 year olds in Wales, England and Scotland, conducted as part of an ongoing evaluation of the impact of the TPD on youth smoking and e-cigarette use. Interviews were conducted around the end of the transitional period for TPD implementation, and explored perceptions of e-cigarettes and tobacco, as well as similarities and differences between them.

**Results:**

Young people differentiated between tobacco and e-cigarettes, rejecting the term e-cigarette in favour of alternatives such as ‘vapes’. Experimental or occasional use was common and generally approved of where occurring within social activity with peers. However, regular use outside of this context was widely disapproved of, unless for the purpose of stopping smoking. Increased prevalence of e-cigarettes did not challenge strongly negative views of smoking or reduce perceived harms caused by it, with disapproval of smoking remaining high. Nicotine use was variable, with flavour a stronger driver for choice of e-liquid, and interest more generally.

**Conclusion:**

The extent to which participants differentiated between vaping and smoking, including styles and reasons for use in adults and young people; absence of marketing awareness; and continued strong disapproval of smoking provides limited support for some of the potential mechanisms through which e-cigarettes may renormalise smoking. However caution over nicotine exposure is still necessary.

## Introduction

Current UK legislation prohibits sale of vaping equipment to under 18′s and mandates registration of nicotine-based products, and full disclosure of ingredients, with the UK Medicines and Healthcare Regulatory Agency (MHRA) (Tobacco & Related Products Regulations, 2016). Regulation implemented via the European Union (EU) Tobacco Products Directive (TPD) in 2016 included restriction on promotion of e-cigarettes and mandated warning information on packaging (Official Journal of the EU, 2014). Within the heavily regulated context, e-cigarettes continue to be accepted, and promoted, for smoking cessation by bodies such as Public Health England and NHS Health Scotland. This is in stark contrast to other countries such as the US, where the Food and Drug Administration (FDA) have supported significant restrictions on e-cigarettes, including bans on certain flavoured liquids (FDA, 2019) and some regions have gone as far as voting to ban e-cigarette sales entirely (BBC News, 2019).

E-cigarette use among UK adult smokers emerged rapidly from 2011, before plateauing from 2013 (West, Beard & Brown, 2018). While use is primarily reported among adult smokers for the purpose of smoking cessation (Patel et al., 2016), concerns have been raised over potential adoption by non-smokers (Farsalinos, 2018), particularly children and young people (Leventhal et al., 2015). Although contested, concerns include the potential for developmental harms from nicotine exposure in e-cigarettes (England, Bunnell, Pechacek, Tong & McAfee, 2015) and, primarily, on the potential for them to renormalise smoking among youth (Hartmann-Boyce, Begh & Aveyard, 2018).

Rates of ever having used an e-cigarette among UK youth have grown rapidly in recent years (Moore et al., 2015). Data from the UK and North America indicate that rates of experimental e-cigarette use among young people have now overtaken those for tobacco smoking (Bauld et al., 2017; DeMissie, Everett Jones, Clayton & King, 2017; Montreuil et al., 2017; Wills, Knight, Williams, Pagano & Sargent, 2015). Some emerging studies indicate longitudinal associations between trying e-cigarettes and subsequently trying smoking among young people deemed otherwise non-susceptible to smoking (Dai & Hao, 2016; Dutra & Glantz, 2014). Willingness to try e-cigarettes if offered by a friend has been associated with subsequent use of e-cigarettes and later smoking initiation (Bold, Kong, Cavallo, Camenga & Krishnan-Sarin, 2017), with initiation and past 30-day tobacco use higher in those who have used e-cigarettes at baseline than never-users (Soneji et al., 2017). Nevertheless, studies also show that few adolescent experimental users of e-cigarettes progress to regular use (Bauld et al., 2017; McNeill, Brose, Calder, Bauld & Robson, 2018), while UK population-level smoking rates have continued to fall to a historical low in young people (SDDU, 2017) despite this co-occurring increase in e-cigarette use (Warner, 2018).

Declines over time in youth smoking have been achieved via a range of mechanisms. Multi-level tobacco control policies have included measures on price and taxation, decreasing visibility through bans on use, advertising restrictions and educational programmes (WHO 2019). Collectively these have created a cultural context in which smoking has become, not just practically more difficult, but also denormalised, with the non-smoking majority collectively stigmatising smoking behaviour (Bell, McCullough, Salmon & Bell, 2010a; Ritchie, Amos & Martin, 2010; Schroeder, 2008). Although controversial (Bell, Salmon, Bowers, Bell & McCullough, 2010b), decreased social acceptability of smoking has been significant in motivating quit attempts in adults (Hammond, Fong, Zanna, Thrasher & Borland, 2006) as well as in declining youth adoption (Malone, Grundy & Bero, 2012). For renormalization to occur, e-cigarettes would need to reverse the effects of the broad range of historical and contemporary actions which have acted to denormalise smoking (Sæbø & Scheffels, 2017). This would require a change in the, now largely negative, social norms around smoking to a re-emergence of more positive attitudes (Voigt, 2015), as well as requiring reversal of the spatial and legal constraints – and public approval of these - that have limited where and when smoking can occur (Collins & Procter, 2011).

Various mechanisms have been suggested through which renormalisation could potentially occur if the presence of such mechanisms was identified in young people. These include: whether the growing presence of e-cigarettes could increase acceptability of smoking through perceived similarity; whether increased prevalence in e-cigarette use could lead to behaviour change through increased acceptance of tobacco (Sæbø & Scheffels, 2017); and whether increased visibility of marketing and branding for e-cigarettes normalises smoking products (Sæbø & Scheffels, 2017; Voigt, 2015). Further debate centres around the nicotine gateway theory, based on concerns over pharmacological responses, including release of dopamine creating a pleasure response, which is desensitised through repeat exposure, thus creating cravings (Benowitz, 2010). Proponents argue that exposure to nicotine through e-cigarettes will therefore lead to seeking nicotine through tobacco (Auf et al. 2016), although this is largely unsupported by current evidence (Bell and Keane, 2014; Hallingberg et al., 2019).

The renormalisation hypothesis makes strong assumptions about the perceived relationship between tobacco cigarettes and e-cigarettes. Hence, understanding how young people perceive these as independent or interacting products, is vital in assessing the theoretical coherence of this hypothesis. Studies have identified that for some young adults who move from smoking to e-cigarettes, they can represent a positive health choice and a form of freedom from smoking (Keane, Weier, Fraser & Gartner, 2017; Yule & Tinson, 2017), signifying a positive relationship with e-cigarettes that may not be evident in younger people who have not previously smoked. To date, there is some evidence that young people perceive e-cigarettes as less harmful than smoking (Amrock, Lee & Weitzman, 2016; Wills et al., 2015) with lower risk perception positively associated with chances of ever using (Montreuil et al., 2017). However, youth support for strong regulation has also been identified, driven by concerns over unknown health risks (Weishaar, Trevisan & Hilton, 2016). Some studies indicate that young people themselves believe that e-cigarettes may be a gateway to later smoking (Akre & Suris, 2017). However, e-cigarette experimentation may be driven by features which are distinct from tobacco cigarettes, for example flavours or other product characteristics (Dai & Hao, 2016; Hilton, Weishaar, Sweeting, Trevisan & Katikireddi, 2016), with such differences potentially contributing to further denormalisation of tobacco smoking.

This study reports results from qualitative research conducted as part of an on-going multi-site, mixed-methods evaluation of the impact of the EU TPD on young people's smoking and vaping behaviour in the UK (Moore et al., 2017). This legislation is an example of regulatory action driven by a conviction that “electronic cigarettes can develop into a gateway to nicotine addiction and ultimately traditional tobacco consumption, as they mimic and normalize the action of smoking” (Official Journal of the EU, 2014). Findings are here discussed in relation to the presence or absence of potential mechanisms through which renormalisation may act.

## Methods

This paper reports findings from semi-structured group interviews taking place between March and July 2017.

### Sampling and participants

Seven schools across Wales, Scotland and England were recruited to represent a range of urban/rural locations and socio-economic compositions. Socio-economic status was identified through levels of pupil entitlement to the provision of free school meals (FSM), which have been confirmed as a reliable measure of socio-economic status (Taylor, 2018). For Wales, where ‘ever-use of e-cigarette’ rates by school from 2015 were available from the School Health Research Network (SHRN) survey (Hewitt, Roberts, Fletcher, Moore & Murphy, 2018), this was used to select across a range of school-level vaping rates. Group work was conducted with Year 10 students in England and Wales and S3 students in Scotland (i.e. students aged approximately 14/15 years), representing a key age for increased smoking in UK youth (De Lacy, Fletcher, Hewitt, Murphy & Moore, 2017). Twenty-one group interviews took place across 7 schools (*N* = 76 pupils, 39 female), comprising small single sex friendship groups of 2–6 young people, from a range of academic abilities. Table 1 Schools information presents school details.

**Table 1:** Schools information.

School code	Location	FSM rate and ever-vaping where available (relative to national FSM registration*)	Number of groups	Total number of pupils
***Wales***				
A1	Semi-rural	Low FSM Medium vaping	4	13 (8 female)
A2	Semi-rural	Medium FSM Low vaping	4	16 (8 female)
A3	Urban	High FSM High vaping	2	8 (4 female)
***Scotland***				
B1	Semi-rural	Medium FSM	2	6 (3 female)
B2	Urban	Low FSM	2	6 (3 female)
***England***				
C1	Rural	Low FSM	4	15 (8 female)
C2	Urban	High FSM	3	12 (5 female)

⁎ National FSM national registration: England 14.3% (Local Government Association, 2016); Wales 18% (Taylor, 2018); Scotland 37.6% (Scottish Government, 2017).

Pupils were not selected on the basis of their own smoking behaviour or susceptibility, and hence provide insights into normalisation processes from the perspectives of a diverse cross section of young people. School staff were asked to explain the study to their classes and identify established friendship groups among those interested in taking part, to maximise rapport between participants. Although group interviews can risk the emergence of conformity of views (Sim, 1998), perceived similarity among members was helpful here in facilitating comment and enhancing exploration of individual and collective views (Tong, Sainsbury & Craig, 2007). Interviewers reported that atmosphere was positive in a majority of groups, with a willingness to engage with the topic. In later debrief, it was suggested that atmosphere reflected the novelty and relatively recent emergence of e-cigarettes into the landscape, meaning that young people were perhaps more interested in them than at the present time. In a couple of groups, discussion was more stilted where expected participant numbers had not materialised due to pupil absence, resulting in two attendees. This was felt to be associated with an absence of peer support and the feeling of being more exposed than when larger numbers are present.

In Wales, schools were recruited through SHRN, with the SHRN coordinator initiating contact with seven schools by email, and follow up by RB. Four schools consented to participate, although the fourth was unable to accommodate data collection within the time frame available and hence participated only in subsequent follow-up data collections not reported here. In Scotland, a research case must be made to the relevant Local Authority (LA), before permission is granted to approach schools. Of eight LAs contacted, permission was granted by three, with two rejections and two non-responses. JM contacted six schools in each of two of the approved areas, with two schools agreeing to participate. In England, ten schools were contacted by OM, with three agreeing to participate and seven non-responses. Group work was completed at two, with the third withdrawing too late for further recruitment.

### Ethics and consent

Ethical approval was obtained from Cardiff University School of Social Sciences Research Ethics Committee on the basis of an opt-in consent process, requiring active approval by parent/carers. This was secured through advance notification and return of a signed consent form before the date of interviews. Pupils were provided with information and the opportunity to ask questions prior to groups and before signing to indicate their own consent.

### Data collection and analysis

A topic guide was developed to reflect study research questions, including the theorised mechanisms of the TPD legislation. This was pre-tested through patient and public involvement (PPI) to enhance relevance and acceptability (Entwistle, Renfrew, Yearley, Forrester & Lamont, 1998). Consultation took place with an existing youth forum with familiarity with research activity (http://decipher.uk.net/public-involvement/young-people/), and the resulting suggestions were incorporated. These included: use of example pictures of e-cigarette devices to prompt discussion on common terminology; prompts on social risk of use as well as health risk; prompts on context of use e.g. social settings.

Final topic guide centred around:

1 perceptions of e-cigarettes in relation to tobacco and as a standalone product
2 interaction with, and awareness of, e-cigarette marketing and health warnings
3 perceived prevalence and perceived risks of e-cigarettes use
4 e-liquid selection and awareness of the presence/absence of nicotine

Audio recordings were transcribed verbatim (excluding hesitations, filler utterances etc.). A coding frame was developed by RB from initial reading of transcripts and themes from the interview topic guide. This was refined through discussion with other members of the research team and a final frame established. All transcripts were then coded by RB, with 20% coded concurrently by JM, and study authors then further refining coding through discussion. This enhances consistency of analysis by reducing interpretation and facilitating exploration of disagreements (Berends & Johnston, 2005). Analysis was approached through a Critical Realist lens, incorporating deductive and inductive elements (Hyde, 2000). The application of critical realism here refers to analysis which draws on existing theory (here on potential mechanisms of renormalisation) while still engaging with participant experiences and understanding of the issue (Fletcher, 2014), to both consider assumptions underpinning the renormalisation hypothesis and to openly explore identified issues. This involved two-phased coding (Saldãna, 2013), with open reading followed by thematic analysis, with themes reflecting the interview topic guide and underpinned by main study aims.

## Findings

Findings are here structured to explore potential renormalisation mechanisms discussed above. Quotes are used throughout as examples relevant to this debate and, as such, have been actively selected by the authors to facilitate this discussion rather than to suggest a ‘typical’ response.

School code, group number and gender are indicated in brackets.

### Perceived similarities and differences between e-cigarettes and tobacco cigarettes

The term ‘e-cigarette’ was widely rejected by participants, with ‘vapes’ and ‘vaping’ preferred in almost all groups. As the preferred terminology of participants, ‘vapes’ and ‘vaping’ will be used throughout the rest of this report. Participants were far more likely to describe the more modern ‘tank’ or ‘pen’ style devices as used by their age group, than to recognise earlier models designed to mimic the appearance of cigarettes (cig-a-likes). There was general awareness of how vapes functioned, including the need to charge them and the production of vapour not smoke.

When asked why young people vaped, reasons included: for fun with peer group, for flavours, and to show off to peers both in real life and online through posting tricks on social media. These, predominantly social reasons, contrasted with perceived reasons for adult use, described primarily as to stop smoking:

*Like, you don't see people our age going round with the ones that look like cigarettes* – *Maybe adults would, the ones trying to get off smoking.* (A2, 1, F).

Several commonalities between vapes and tobacco cigarettes were described, such as potential addictiveness, nicotine content, and potential to be harmful. Stated differences were however, more extensive, with non-nicotine e-liquid options and range of flavours most commonly referenced and vaping defined as different to smoking by a majority. Developments in technology, including different styles of device and range of flavours, appeared to enhance their distinctions from tobacco cigarettes.

In discussing perceived risks of vaping we encountered more variation in responses within groups than for other areas such as perceived prevalence, with peers often disagreeing over risks or stating something that others were unfamiliar with, leading to discussion over the validity of such claims. This included disagreement over whether they were addictive, contained nicotine and caused illness such as cancer. This appeared to be driven by absence of clear messaging on vaping in contrast with smoking. While smoking harms are taught in school from an early age and are accompanied by consistent public health information, this was not the case for vaping, with none reporting that their school had covered this and frequent reference to getting information – or misinformation – from social media. There was also notable variation in awareness of the legal age for vaping in the UK, again potentially reflecting the absence of reliable information encountered.

The types of risks also often varied from those of smoking, with health risks such as cancers and respiratory conditions significantly less likely to be mentioned than for smoking. For vaping, risks commonly focussed on anecdotes of operating or charging problems such as devices ‘blowing up’ (again, frequently citing stories from social media), and unknown toxicity of the chemicals in liquids.

Although clearly viewed as less harmful than smoking by most, this ‘hearing differently’ was also evident:

*Some people say they have even more…they claim to be less harmful but actually they're more harmful in fact, apparently.* (A1, 4, F)

It was common to express caution that harms may yet be discovered in the future, as had happened with smoking:

*It's just with e-cigarettes, you are not sure what's bad about them. Like, you know some of them are nicotine. So it's just, what health impacts? Smoking, you know that you can get lung cancer, but e-cigarettes there isn't the same health issues.* (B1, 2, F)

### Perceived prevalence of vaping and impacts on smoking norms

Groups were encouraged to share their own experiences of smoking and vaping but were advised that they did not have to do so. Many did discuss their own experience of trying vaping and this often occurred at group-level e.g. all in a group had tried or none had, likely reflecting the selection of established friendship groups as participants, where shared norms and behaviours are more likely. Being a regular vaper was rarely discussed but emerged in two male groups, where members reported regularly vaping non-nicotine liquids to do tricks. This patterning by group was not the case for smoking, with much lower numbers stating that they had tried it and just a few individuals stating that they were regular smokers.

Prevalence for both vaping and tobacco smoking were explored through discussion of perceived rates of smoking/e-cigarette use among young people and adults, as well as changes in recent years. Most agreed that youth vaping had increased in the last few years. Some suggested that there were still more young smokers than vapers, with this particularly observed in groups at one school in England (C1), where smoking was described as growing recently. However, more felt that they saw more young people vaping than using tobacco cigarettes now.

Vaping was largely described as casual, or occasional, and strongly socially motivated, for example occurring with peers at parties where a device is brought by one person and passed around. Although this was common, many also suggested that vaping experimentation was a fad or trend that had peaked and was now reducing in both use and interest in people their age:

*Not too long ago there was like a whole new craze about it because everybody was thinking it would be a safer alternative to smoking and ever since then the boom of it has started to die down and we haven't really seen much of anymore.* (C2, 1, M)

Regular use (i.e. daily or weekly) by young people was observed as rare, adopted as either a means to perform tricks (where nicotine content of liquids was unimportant) or as a more habitual behaviour associated with smoking. Habitual users were described as more likely to either be dual users of tobacco and vapes or at least to be members of smoking peer groups. It was also frequently stated that this group were more likely to be engaged in alcohol and cannabis use, with regular vaping perceived as a component of a broader clustering of risk behaviours. Regular smokers were described in negative terms, as the *‘kind of disruptive ones’* (C1, 3, F), or as *‘people who think they're hard’* (B1, 2, F) and to be seen as less academically engaged:

*I don't think it's the more intelligent people that smoke.* (A2, 4, M)

Some also suggested they were likely to use cannabis as part of their identity as smokers:

*…a lot of them will just smoke any of them, anything they can get their hands on.* (A3, 1, M)

Increased prevalence of casual vaping, including popularisation of tricks (creation of shapes with vapour clouds) among both peer groups and on social media, was described as making experimental use acceptable as well as reducing concerns over associated risks. ‘Having a go’ on a vape as a social activity was met with little disapproval, with reasons for experimental use cited as (i) because peers were doing so, (ii) for flavours (including swapping flavours), (iii) for doing ‘tricks’. However, many participants differentiated between acceptance of casual vaping, but disapproval of regular use unless as an aid to stop smoking. Regular use was otherwise pointless and, in some cases, ‘not cool’:

*If you're just doing it to be sociable and pretend, and say, “Yeah I'm vaping,” and just blowing out, you're just going to be bullied, not like bullied constantly, but you're going to be taken the mick out of you.* (C1, 2, F)

An exception to this was regular vaping to perform tricks:

*I think most people if you're going to vape you've got to do tricks with it in our year because if you're not doing tricks you're not cool.* (C1, 2, F)

Throughout the data, there was very little variation by gender for most themes but an area where this occurred was in consensus in most groups that boys were more likely to be involved in trick performance, and a small number of non-smoking boys discussed this, including sharing through social media, cited as very common, with those who did so confident of little censure from peers:

*You see some people sharing videos on Facebook and they're having a big puff of this e-cigarette and they're making rings with it and it looks cool, it's almost like a new kind of trend.* (A2, 4, M)

Perceived peer and family disapproval of regular smoking or vaping was strong. When discussing expected peer reactions to them becoming smokers, responses included the expectation of respect for their choice: *‘…if you want to do them just do them’* (C1, 4, B), to the, more common, expectation of negative responses, from concern over addiction:

*I know my friendship group would say like, what are you doing? Just stop it now while you've got the chance.* (A3, 1, F)

to a reaction closer to disgust:

*I think with my friends it would repel them*. (A1, 4, M)

Potential risk of social loss due to exclusion from peer groups was often described as a consequence of becoming a regular cigarette user, but far less evident for regular vaping.

Shared norms within friendship groups were evident and tended to manifest in the relative acceptance of vaping across all in the group or the shared rejection, with some groups keen to identify themselves as not being the ‘types’ to do that.

Most group discussions suggested that tobacco smoking by young people was driven by either addiction or a deliberate adoption of risk (e.g. being done ‘to look hard’):

*…because they think it* (tobacco) *makes them look cooler because it can harm you.* (A2, 2, F).

In several groups it was suggested that rather than making smoking more acceptable, the increased availability of vaping actually made smoking seem more socially unacceptable by comparison, because there is now a less harmful alternative:

*I think with the introduction of e-cigarettes, I mean tobacco was already considered pretty dangerous but I feel like people are being more sceptical about tobacco use and stuff, and e-cigarettes… I think it kind of makes us think that tobacco is more dangerous than it was before.* (C2, 1, M).

All stated that parents would have a worse reaction to smoking than vaping, although parents reacting badly to the latter was also reported by many and, again, tended to be consistent in groups. Some suggested, however, that parents may be less anxious about vaping than tobacco smoking:

*I think they would prefer vaping to smoking, I would say, if it had to be one.* (A1, 2, F)

Reasons for strong parental disapproval of smoking included risks being so well established that ignorance of harm could not be a defence, and decreased peer influence to smoke due to lower prevalence and acceptability in young people:

*I don't think they'd see any excuse for tobacco cigarettes because there's no pressure to have them, there's no influence*. (A1, 1, M)

Some, particularly in those groups keen to identify themselves as not being the type to vape, further expressed concern that parents perceived a greater alignment with drug use for tobacco and were more likely to fear that they were involved in other drug use, such as cannabis, if they were smoking tobacco than if they were vaping:

*I think that after that they will just think worse of us, like they think we'd be doing drugs and stuff like that because of that one thing (tobacco).* (A2, 2, F).

### The impact of device characteristics and vape marketing

There was general agreement within and across groups that vape were easily obtained, and more accessible than tobacco cigarettes. A notable exception to this was in school C1 in England where, as reported above, participants also felt that smoking was more common than vaping. Young people predominantly discussed availability through online sales or informal school supply chains. Within-group discussion was common here, with participants seemingly enjoying sharing stories of who in or around school may be the one selling supplies. It was not uncommon for the same name to come up in multiple groups in a school, suggesting common knowledge of who would *‘sell it on’* (C1, 3, F).

Although some cited pupils in school selling tobacco cigarettes, this was less common, with tobacco largely expected to come from older people supplying it, including peers/siblings and proxy purchasing. The dominance of this informal supply line meant that a majority of pupils who had seen vapes had only seen them outside of the packaging, meaning brand awareness was extremely low. While most were aware of specialist shops in their area or the nearest larger town, few knew these by name or saw them as a likely source of supply, with informal and online routes preferred and seen as less restricted. Several cited that vaping had come into school first through the ‘*populars*’ (A1, 4, F), and spread as a casual and socially-driven practice, including sharing flavours and performing tricks. This contrast with the more deviant status of smoking – and smokers – to many. Flavours were highly significant, both for taste and for the social gains of swapping and lively within-group discussion was common here regarding who had tried which flavours and which were preferred:

*I just liked the different flavours.* Cos *my friend had jam donut, another friend had gummy bear flavour and Heisenberg which is a minty flavour which is quite nice.* (B1, 1, M).

Flavours further differentiated younger, casual users from both adult vapers and from tobacco smokers:

*Because they're always shown with all the flavours and I think if you were trying to quit smoking you wouldn't be bothered by all the flavours.* (A1, 1, M).

It was common to discuss vaping devices as fashion accessories, with appeal associated with the style and cost of equipment:

*I think what makes it more appealing, there are some designs on them. So people are just like, “Oh that's a cool design.” And different flavours and they're comparing with their friends. Oh look at yours, yours is red and mines like camouflage.* (A2, 4, M).

For the few who reported vaping for tricks, device type was also important including use of ‘bigger boxes’:

*I think our age group normally go for them because you can make a fat cloud out of them.* (C1, 1, M)

This sense of using as showing off financial status and being started by more popular pupils contrasted starkly with comments on the typology of tobacco users who, as noted earlier, were commonly defined negatively, including as being the scruffy/naughty/disruptive pupils who were already likely to reject school rules and behavioural norms.

### Attitudes towards nicotine in vapes and potential pathways to tobacco use

Groups were first asked to discuss similarities and differences between vapes and tobacco cigarettes, highlighting widely varying understanding of the presence or absence of nicotine in e-cigarette liquids. Some were unsure or unaware that nicotine could be present and were surprised when others in the group highlighted this, stating that they had thoughts liquids were just:

*…syrupy stuff in different flavours.* (C1, 3, F)

When asked to describe reasons why young people smoke, addiction to nicotine was frequently cited but not so in response to the same question for vaping, where social reasons such as fitting in and showing off where dominant. However, when asked to identify potential risks of vaping, addiction was cited by at least one participant in most groups, suggesting that fear of nicotine dependency was common.

While some stated that they would avoid nicotine-based vaping liquids due to fear of addiction, for many of those who described experimental vape use, nicotine content of e-liquids was either an unknown, or less important than flavour:

*I don't know. It's not really something you ask if its nicotine or no nicotine. You more just ask them what kind of flavour is it* (B1, 2, F).

This reflects the context of use, where the vape belonged to someone else and was passed around for people to try rather than being something actively sought. Where vapes were tried once with little intention of using again, concerns over nicotine were secondary to social factors driving experimentation:

Int: Yeah, so just once?

P: Yeah.

Int: And do you know if that had nicotine or no nicotine in it?

*P: I have no idea [laughs].*                 (C1, 3, F)

Where users are unaware of nicotine content of liquids, this carries risk of developing nicotine withdrawal which may drive further use. However, although it is necessary to be cautious over unintended nicotine exposure through ignorance, there was little evidence of deliberate pursuit of nicotine as a driver for vaping and some evidence of fear of addiction – as well as fear of social judgement of regular use - as a deterrent to more frequent access.

## Discussion

It is arguable that for tobacco renormalisation through use of e-cigarettes to occur, there would need to be a softening of legislative changes that have made smoking more difficult, as well as of the social denormalisation (Hammond et al., 2006) and stigmatisation of smoking (Voigt, 2015). This study explored the presence or absence of potential mechanisms that may reverse denormalisation through qualitative discussion with young people who are growing up in a legal and politically hostile environment for smoking.

The young people in this study highlighted multiple differences between tobacco cigarettes and e-cigarettes, including availability of flavours and non-nicotine liquids, which were significant in use among their age group. Likewise, young people in this study clearly rejected the term ‘e-cigarette’, instead endorsing names which differentiated these products from tobacco cigarettes. This differentiation may suggest that, despite rapid growth, vaping could act to further denormalise smoking through divergence of device types which ensure it is recognisable as a non-smoking behaviour.

Varying attitudes towards vaping among young people have been previously associated with a void in formal information (Lucherini, Rooke & Amos, 2018) and this was also suggested here, with variation in views of the harms of vaping in the absence of information from either school or public health sources. Clear messaging from public health bodies on health risks and on the positioning of vaping as a smoking cessation aid, may be helpful as both direct communication and in aiding schools in developing content for pupils.

We identified that, although it is arguable that trying vaping had become normalised and was subject to relatively positive social norms among participants, this was confined to experimental use in their age group and stopping smoking in adults. Previous research has identified positivity among older ex-smokers towards vaping as a means of liberation from smoking (Keane et al., 2017; Yule & Tinson, 2017), while being able to continue the performative aspects of smoking in a more acceptable way (Lucherini, Rooke & Amos, 2018). This was not echoed here, suggesting that mechanisms of acceptability may function differently in young people who have not developed this understanding of the role of vaping from their own smoking experience. This apparent weaker attachment to vaping than is found in older ex-smokers supports findings that young people are unwilling to be seen replicating the performance of smoking through vaping (Lucherini, Rooke & Amos, 2018), and suggests that proposed pathways to renormalisation may vary by population. Where positivity towards vaping was displayed, this did not extend to smoking, with strong disapproval throughout, as well as disapproval of regular vaping.

Findings echoed previous research highlighting the value of sharing to young people, as well as the performative aspects of tricks (Katz, Erkinnen, Lindgren & Hatsukami, 2019; Measham, O'Brien & Turnbull, 2015), associated with increased visibility of newer style devices replacing older ‘cig-a-like’ models. These served to further differentiate vaping from smoking, suggesting that neither similarity nor increased visibility were present as renormalisation mechanisms among this sample. The absence of brand knowledge, the dominance of peer to peer routes and the stylistic differences between vaping equipment and tobacco cigarettes suggests increased visibility as an unlikely mechanism for smoking renormalisation.

Trying e-cigarettes was discussed as normalised in youth behaviour and observed here within peer groups, where it was common for friendship groups to have experimented together and mirroring US findings on trying vaping as a group activity where devices are passed around (Alexander, Williams & Ok Lee, 2019). This was primarily as a casual behaviour driven by appealing flavours rather than a habitual one and seemingly of decreasing interest, supporting findings on the plasticity of use of these devices (Katz et al., 2019^),^ and congruent with the quantitative trend analysis conducted in the wider study (Hallingberg et al., 2019). Evidence suggests that the majority of regular vapers are tobacco smokers (Bauld, MacKintosh, Ford & McNeill, 2016), and that was supported here, with very limited indication of any overlap between casual vapers and smokers, and regular use seen as a marginal behaviour engaged in by smokers (who were themselves marginalised and negatively viewed). It is notable that regular vaping was still largely disapproved of, although less so than for regular smoking. In terms of acceptability, there was no evidence of increased approval of smoking in relation to increased prevalence of vaping, and therefore no suggested mechanism here for smoking being renormalized through increased positive perception leading to changing social norms. It is arguable that increased visibility of vaping has normalised trying vaping rather than trying smoking (Britton, Bogdanovica, Ashcroft & McNeill, 2014), however, although the frequency of casual vaping identified did not appear to translate to approval of smoking, absence of longitudinal data means that later smoking adoption cannot be discounted.

Previous findings have suggested that youth vaping is often motivated by social and sensory experience rather than nicotine (Pokhrel, Herzog, Muranake & Fagan, 2015) and this was evident here, with flavours generally more important than nicotine effects. However, young people reported that supply chains for vapes within their schools centred on informal supply via peers, leading to cases where they reported being unaware of the nicotine content of liquids they had experimented with. This, coupled with apparent normalisation of vaping experimentation described both here and elsewhere (Britton et al., 2014), suggest some risk of inadvertent exposure to nicotine creating a pharmacological pathway to repeat use (Benowitz, 2010). However, absence of evidence of repeated and regular use of vaping, as well as few accounts of progression from vaping to smoking, suggests that exposure to nicotine was not here creating a gateway into nicotine addiction.

Findings support previous studies that suggest dual vape and tobacco use is associated with higher rates of alcohol and other drug use (De Lacy et al., 2017; DeMissie et al., 2017; Dunbar et al., 2017), suggesting that regular vaping may form part of deliberate risk behaviour in a minority of young people. Co-occurring use may also be associated with increased tobacco consumption in smokers (Doran et al., 2017), suggesting that the potential harm reduction benefits intended through vaping adoption may not be experienced by young regular users. Further investigation is needed into both the rejection of vaping by young smokers and also dual use, to understand how patterns of consumption interact.

### Strengths and limitations

As with all qualitative research, the value of findings lies in the insights generated from participants rather than attempts at representativeness of a wider population. Further exploration of the presence or absence of potential mechanisms of renormalisation across different populations, and in contexts with different political and legislative approaches to smoking, would add to the insights and interpretation presented here. It is also noted that these perspectives were gathered at one point in time from a cross section of young people, and so cannot capture the fluctuations in behaviour and attitudes that are likely/possible within adolescence. Further longitudinal research is therefore recommended.

Although engagement with the topic was felt to be high by all interviewers, it must be acknowledged that self-report occurs within social and cultural contexts which may impact responses (Razavi, 2001). This may include reluctance to disclose an illicit behaviour, such as underage vaping, to an unknown adult. As vaping was found to be widespread here and, at this time, a seemingly-popular new trend, social desirability bias and pressure to appear interested and knowledgeable, may have also been a factor.

Although no claims of representativeness are made it is noted that, as a condition of ethical approval, the study utilised opt-in parental consent processes for groups participants. There is some evidence to suggest that such methods can produce bias through over-representation of adolescents from lower risk populations (Shaw, Cross, Thomas & Zubrick, 2014), as well as lowering response rates (Junghans, Feder, Hemingway, Timmis & Jones, 2005). Although sampling incorporated a range of locations and socio-economic groups, this should be considered in interpreting findings.

The study benefitted from the inclusion of PPI to test and refine the topic guide. This consultative process ensured that there were no barriers associated with terminology and increased acceptability of discussion topics. Data was obtained from three UK nations, representing a broad range of geographical locations and socio-economic groups, with consistent identification of themes across schools supporting the strength of findings. The use of second coders for the development and application of coding framework further ensured that analysis was consistently applied across the data set.

### Conclusion and implications

Vaping was not viewed here as synonymous with tobacco, and hence, the growing visibility of vaping was not softening attitudes to smoking. This, coupled with continued legal constraints to smoking which likely reinforce negative attitudes in those who have grown up with smoking prohibitions, did not suggest significant risk of re-emergence of tobacco use in these young people. While some have suggested mandated name change from e-cigarettes to vapourisers or nicotine control products (Royal Society for Public Health, 2015) to enhance the differentiation between smoking and vaping, changes to the appearance of devices and the ubiquity of the term ‘vaping’ in public discourse, marketing and policy, our research suggests that this differentiation is already occurring. The distinctions made between casual and regular vaping, as well as between vaping and tobacco smoking, do not suggest that the former is seen as similar enough to smoking to trigger uptake among those who would not otherwise have smoked.

While casual vaping appears to have become normalised in youth culture, smoking (and regular vaping) continues to be characterised negatively and widely disapproved of by young people. Casual vaping was rarely described as being driven by nicotine use, though the nature of informal supply chains described by young people does suggest that some young people may be unknowingly exposed to small amounts of nicotine. The dominance of these supply chains has implications for efforts to limit youth access, as disrupting these routes is not likely to be achieved by top-down regulation which may actually exacerbate the issue.

These qualitative insights add to other recent research on vaping and smoking amongst young people in the UK (Bauld et al., 2017; Hallingberg et al., 2019) which suggest that experimentation with these devices is common, but regular use is largely confined to young people who smoke, with youth smoking rates in the UK continuing to decline at an encouraging pace. Overall, among participants selected to reflect a key age for smoking initiation, potential mechanisms for smoking renormalisation were not present. Although vaping use may present some risks in its own right, increased likelihood of tobacco smoking as a result was not suggested.

## Declaration of Competing Interest

The authors declare no competing interest.
